# The accuracy of frozen section analysis in ultrasound- guided core needle biopsy of breast lesions

**DOI:** 10.1186/1471-2407-9-341

**Published:** 2009-09-24

**Authors:** Andreas H Brunner, Thomas Sagmeister, Jolanta Kremer, Paul Riss, Hermann Brustmann

**Affiliations:** 1 Department of Obstetrics and Gynecology, Division of Gynecology and Gynecologic Oncology, Landesklinikum Moedling/Vienna, Austria; 2 Department of Pathology, Landesklinikum Moedling/Vienna, Austria

## Abstract

**Background:**

Limited data are available to evaluate the accuracy of frozen section analysis and ultrasound- guided core needle biopsy of the breast.

**Methods:**

In a retrospective analysis data of 120 consecutive handheldultrasound- guided 14- gauge automated core needle biopsies (CNB) in 109 consecutive patients with breast lesions between 2006 and 2007 were evaluated.

**Results:**

In our outpatient clinic120 CNB were performed. In 59/120 (49.2%) cases we compared histological diagnosis on frozen sections with those on paraffin sections of CNB and finally with the result of open biopsy. Of the cases 42/59 (71.2%) were proved to be malignant and 17/59 (28.8%) to be benign in the definitive histology. 2/59 (3.3%) biopsies had a false negative frozen section result. No false positive results of the intraoperative frozen section analysis were obtained, resulting in a sensitivity, specificity and positive predicting value (PPV) and negative predicting value (NPV) of 95%, 100%, 100% and 90%, respectively. Histological and morphobiological parameters did not show up relevance for correct frozen section analysis. In cases of malignancy time between diagnosis and definitive treatment could not be reduced due to frozen section analysis.

**Conclusion:**

The frozen section analysis of suspect breast lesions performed by CNB displays good sensitivity/specificity characteristics. Immediate investigations of CNB is an accurate diagnostic tool and an important step in reducing psychological strain by minimizing the period of uncertainty in patients with breast tumor.

## Background

The widespread mammography screening program for early detection of breast cancer gave rise to a large number of biopsies taken in order to determine the nature of sono- or mammographically diagnosed breast abnormalities. In the diagnostic concept, percutaneous image- guided CNB has become an alternative to fine needle aspiration cytology (FNAC) and to the open surgical biopsy. FNAC is a relevant test, mainly feasible for cystic lesions [1], surgical open biopsy is more invasive [2] and expensive [3].

Ultrasound- guided automated core biopsy was first described by Parker et al in 1993 [4]. Since that time, other investigators have also demonstrated that ultrasound- guided 14- gauge automated core biopsy is safe, fast, accurate, and costsaving [2,5,6]. Thin frozen sections of excellent histologic quality can be prepared in a matter of minutes from virtually any non- calcified fresh tissue excluding fat. Because of this, its application in the evaluation of breast lesions is excellent, being limited only by the fatty content of the biopsy material. In the case of infiltrating breast carcinoma, not only can one establish the accurate diagnosis but other observations, such as histologic subgroup and the degree of differentiation can also be obtained.

In this article we briefly review the results of US- guided CNB and frozen section analysis of the specimens in order to require a rapid diagnosis and to reduce psychological strain by minimizing the period of uncertainty in patients with breast tumor. The time needed for diagnosis represents a major period of anxiety for the woman. Providing a definitive diagnosis within a short time reduces patient stress [7,8]. There is some promising evidence from earlier published studies which are focused on this topic yielding an excellent reliability [9-11].

The aim of the present study was to provide data on the accuracy of US- guided CNB and frozen section analysis comparing histological diagnoses made on frozen section specimens of the CNB with those made on paraffin sections of CNB and definitive results of tumorectomy specimens in patients with breast lesions.

## Methods

We examined medical records of 109 consecutive patients with palpable or non- palpable breast mass, which were treated with an automated biopsy gun (Bard Magnum™) and 14 gauge needle(Ultracore™, inter.^® ^Medical Device Technologies Inc., Gainesville, Fl, USA) under handheld ultrasound guidance with a 12 MHz small parts probe (Logiq 5, GE Medical System, Milwaukee, Wisconsin, USA). All patients were treated in the Department of Obstetrics and Gynecology at the Landesklinikum Thermenregion Moedling/Vienna, Austria. The institutional board approved this retrospective data analysis.

During a period of 24 months, two- dimensional (2 D) US- guided biopsies were taken from 109 patients referred to our clinic with a total of 120 palpable or non- palpable breast lesions that had been initially detected by palpability, mammography, and/or ultrasound imaging. No initial biopsies by surgical excision, stereotactic biopsy, or any other means were performed of those lesions before US- guided CNB. We performed a 2D- ultrasound examination of the patients in supine position and elevated arms to localize the primarily detected or additional lesion in the same or contra lateral breast. Prior to the first breast biopsy, after detailed explanation of the procedure, an informed consent was obtained and a history of blood coagulation problems was requested from each patient. No laboratory tests were performed unless the patient was under anticoagulation therapy or reported a history of coagulopathy.

**Figure 1:**
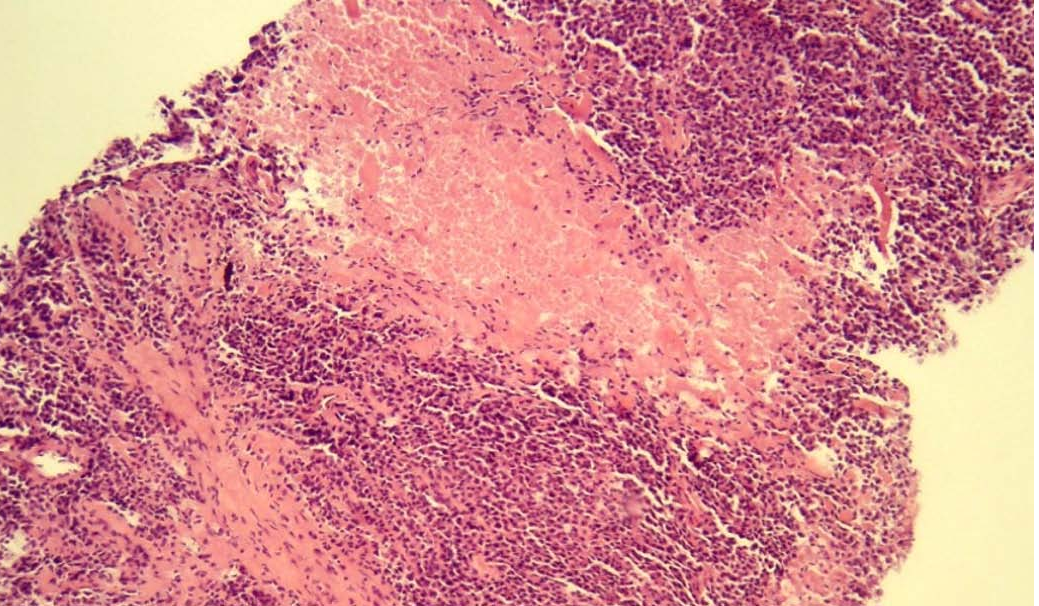
**Breast core biopsy showing infiltrating ductal carcinoma (frozen section, ×100)**.

All biopsies were performed by means of a core needle throw with 22 mm excursion. In order to achieve uniformity of methods, positioning of the CNB needle was performed exclusively by three persons who had undergone dedicated training in CNB techniques and mandatory performed open biopsy if necessary. The procedure was carried out strictly according to a standard protocol described previously [12]. The patients skin was prepared with antiseptic solution and local anaesthetic applied. After the needle has been placed at the edge of the lesion under 2D- US guidance, the 22 mm core needle throw was executed. The passage of the needle through the lesion could be directly visualized and confirmed. The number of cylinders of tissue varied from one to four, if feasible one cylinder was sent to frozen section analysis. All frozen sections specimens were analysed by two board-certified pathologists, specialized in gynaecological pathology. Frozen section pathologic examination was performed according to a specific protocol. Figure 1 shows a representative example of CNB and staining in frozen section analysis. After a median time of 14 min the preliminary histological diagnosis was available. The remaining tissue was routinely stained for permanent paraffin embedded preparations. Architectural structure of the lesion, steroid receptor content, tumor grading, p-53 and the estimation of HER-2 over expression were ready for diagnostic work up between the next 24- 48 hours. Overall and parameter- specific diagnostic yields were assessed and comparison between CNB specimen of frozen section analysis and definitive histological finding at CNB specimen or excision biopsy was assessed.

Sensitivity, specificity, PPV and NPV of the frozen section analysis by CNB technique were calculated. Values are given as medians (range), means (standard deviation [SD]) or absolute numbers. Metric measures were compared using Mann-Whitney U test, Fisher's test or chi- square tests were used in case of categorical variables, as appropriate. *p *values < 0.05 were considered statistically significant. We used statistical software SPSS 11.0 for Windows (SPSS 11.0, SPSS Inc., Chicago, IL) for statistical analysis.

## Results

In our series of 109 patients, 11 women underwent multiple CNB, therefore 120 breast samples were investigated. The median age for all patients was 63 (range 33-98) years. 82 women were after, 27 were before the menopause. The median ultrasonographic lesion size was 15 mm (range 4- 60 mm), 63 tumors were palpable, 57 were non palpable by clinical finding. The ultrasonographic findings of the 120 lesions were mass in 101/120 (84.1%) and non-mass in 19/120 (15.2%). Of the cases 67/120 (55.8%) were proved to be malignant and 53/120 (44.2%) to be benign, thus the ratio of benign to malignant biopsies was about 1.2: 1 (Table 1).

**Table 1:** Patients' characteristics with breast lesions and CNB.

Parameter	Value*
Total number of patients enrolled	109
Total number of biopsis enrolled	120
Age at diagnosis (years)	62 (33-98)
Multifocal/Bilateral lesions	11
Postmenopausal status	82
Ultrasonographic lesion size (mm)	15 (± 10)
Non palpable tumor	57
Positive lymph node status	25
Malignant finding at paraffin section	67

*values are given as absolute numbers, mean (± standard deviation) values, or median (range) values; CNB = core needle biopsy

61/120 (50.8%) CNB of breast lesions were evaluated only by paraffin sections. In these cases less material was obtained due to small lesion size or poor quality of the CNB specimens and material was saved for routine paraffin embedding (Table 2). In 59/120 (49.2%) cases we compared histological diagnosis on frozen section technology and those on paraffin section of CNB and finally all of them with the results of open biopsies. Among the 59 cases investigated by frozen section analysis 40/59 (67.8%) were malignant, 17/59 (28.8%) were judged benign by the pathologist. In 2/59 (3.3%) cases frozen sections were unsatisfactory and the pathologist did not commit during frozen section, these lesions were final classified benign. 2/59 carcinomas (3.3%) were only diagnosed on paraffin sections of CNB material, but were missed in the frozen section examination (Additional File 1). This was due to wrong positioning with folding of the CNB, in one case; in the other case a mucinous carcinoma was found in a separate section of the CNB, which was examined in paraffin section slides only. No false positive findings of frozen section analysis were encountered. There was no further discordance between paraffin section of CNB and the final result of the histological diagnosis of the subsequent open biopsy. In 2/59 (3.3%) cases the pathologist found invasive lobular carcinoma instead of invasive ductal carcinoma by diagnostic work up. On frozen section sensitivity was 95%, and the PPV was 100%. Full specificity was 100%, and the NPV was 90% (Table 3).

**Table 2:** Patients' characteristics with breast lesions and CNB.

Parameter	Frozen section and paraffin section analysis (n = 59)	Paraffin section analysis only (n = 61)
Sonographic lesion size (mm) *	19 (± 10)	13 (± 10)
Age at diagnosis (years) *	62.25 (± 15.28)	61.63 (± 15.71)

*values are given as mean (± standard deviation) values; CNB = core needle biopsy

**Table 3:** Calculation of frozen section analysis and CNB.

Parameter	Value
Sensitivity	95%
Specificity	100%
PPV	100%
NPV	90%

CNB = core needle biopsy, PPV = Positive prediciting value, NPV = Negative prediciting value

In univariate analysis the investigated tumor characteristics, i.e. tumor histology (*p *= 0.4), tumor grade (*p *= 0.5), tumor size (*p *= 0.4), estrogen receptor content (*p *= 0.3), progesterone receptor content (*p *= 0.3) and Her-2 over expression (*p *= 0.6) did not influence the accuracy of frozen section analysis of the CNB. In addition the patients age (p = 0.08), non-palpable lesions (*p *= 0.6) and the lymph node status (*p *= 0.4), had no relevance for the accuracy of frozen section analysis of the CNB and the final paraffin section result of the subsequent open biopsy. Of note, the results of the statistical analysis are limited due to the small cohort of patients enrolled.

In cases of malignancy (67/120; 55.8%) we obtained the time interval from diagnosis by CNB and definitive treatment. The average interval between diagnosis and treatment by frozen section (42/67; 62.6%) analysis and solely paraffin section analysis (25/67;37.4%) was 13.1 and 11.8 days, respectively (*p *= 0.6). This interval was substantially shorter than that for definitive treatment of benign conditions (73 days).

No clinically significant complications occurred in this study. Cases of minor interstitial haemorrhage, ecchymosis, or self- limiting inflammation were not considered to be significant complications.

## Discussion

Percutaneous ultrasound- guided automated core biopsy is an alternative to surgical biopsy for the histological assessment of breast lesions [13,14]. Frozen section examination of breast core biopsy specimens is an acceptable technique in the initial evaluation of suspect breast lesions [10,11].

In our present analysis of 109 women, we performed 120 CNB. Out of the cases we obtained 59 frozen section analysis and found 2 false negative results yielding an underdiagnosis and overdiagnosing in 3.5% and 0%, respectively. Our reported data are in accordance with previous reports [10,11]. In the early 1980s Gonzales et al [10] compared the results on frozen sections of Tru-cut^® ^needle biopsies in 162 cases in a six year period. There were 20/103 (19.4%) false negative cases of carcinomas in frozen section analysis, one biopsy was considered positive in frozen section but permanent preparations revealed a very atypical intraductal papilloma, yielding a sensitivity and specificity of 77% and 86%, respectively. To our knowledge since then only one paper was published on the accuracy of frozen section analysis in needle biopsies of breast lesions: Mueller- Holzner et al. [11] analysed in a retrospective study the results on frozen section analysis in 2619 cases over a 10 year period. Using a comparable procedure with an automated biopsy gun, they found 1276 malignant lesions. There were 7/1276 (0.5%) false negative cases in frozen section and 5/1276 (0.4%) false negative cases in paraffin section analysis of the CNB. Of note, they also found one false positive case in frozen sections and one in paraffin sections, yielding an overall sensitivity and specificity in frozen section analysis of 99.5% and 85.9%, respectively.

We aimed to find out some risk factors for the cases of false negative results, but we could not demonstrate any clinical or pathologic criteria, which were significantly related. Of note, the limitation of our study is beside the retrospective design, the relatively small cohort of patients for statistical analysis. But on the other hand we could not establish an independent predictor (e.g. tumor histology, tumor grade, tumor size, estrogen receptor content, progesterone receptor content, Her-2 over expression, age of the patients, bilateral disease or the lymph node status) for women with suspect breast lesions for whom the method of CNB and frozen section analysis could not be recommended. The reason for rereading the two false negative cases in frozen section analysis of the CNB was one mucinous carcinoma, which was found later in deeper paraffin section slides and a wrong orientation with folding the core biopsy specimens, respectively.

Based on data showing no sufficient relation between the number of cores and diagnostic accuracy we did not analyse the amount of tissue obtained by CNB in our database [15].

Our study also has strengths, namely the CNB needle procedure was performed exclusively by three persons who had undergone dedicated training in CNB techniques and all frozen sections specimens were analysed by only two board-certified pathologists, specialized in gynaecologic pathology.

From the clinical point of view there is an interesting aspect in cases of malignancy. There is no significant difference according to the time interval between diagnosis by CNB and definitive treatment whether CNB was analysed by frozen section (13.1 days) or solely paraffin section (11.8 days). Taking this into account one can assume that it is possible to reduce the time interval for further one or two days. In this time interval our patients were staged according to a modified staging system for breast cancer including abdominal- and thorax computed tomography, radio nuclide bone scan, gynecologic examination and gynaecologic examination and assessment of serum tumor markers. Thereafter all women were scheduled for surgical therapy or neoadjuvant treatment.

From the critical point of view our procedure did not lead to faster definitive surgical care. With the knowledge of our data, we currently try to improve the procedure in our institution. Now most of the diagnostic metastatic tests are done after the surgery and we consequently aim to shorten the delay to definitive treatment.

In order to avoid false diagnostic cautions, upfront selection of the specimens is needed to identify those that are appropriate for frozen section examination. For example papillary lesions are better classified after multiple paraffin sections are carefully studied [16]. Furthermore, limitations in calcified lesions are recognized due to geometry and histological heterogeneity. For microcalcifications this technique is not feasible and stereotactically- guided CNB is usually preferred since these lesions cannot usually be visualized by ultrasound [17].

## Conclusion

Frozen section examination of breast biopsy specimens has the advantage of high reliability with an acceptable low percentage of false- negative results and practically no false- positive diagnoses. The results are available quickly, the diagnostic information is not limited to 'malignant' or 'negative' but additional information regarding tumor type and degree of differentiation may also be obtained. It is of considerable importance that it is an office procedure saving time and money. The procedure is simple and safe without significant morbidity. Definitive treatment can be discussed and scheduled. It has to be kept in mind that the time of not knowing is associated with major psychological distress and anxiety. Our major goal was to reduce the period of uncertainty between the discovery of a breast tumor and histological diagnosis. From our experience patients highly appreciate the one step procedure.

In summary, we demonstrated, frozen section specimens of CNB under US validation are sufficient to obtain a quick and reliable histological diagnosis of breast lesions.

## Abbreviations

CNB: core needle biopsies; PPV: positive predicting value; NPV: negative predicting value; FNAC: fine needle aspiration cytology.

## Competing interests

The authors declare that they have no competing interests.

## Authors' contributions

AB was critically involved in designing the study, performed the most of the biopsy procedures and drafted the manuscript. TS and JK were involved in performing the other biopsy procedures and were contributed in acquisition of data. PR critically revised the manuscript. HB carried out the most of the frozen section analysis, helped with statistical analysis and was substantially involved in drafting the manuscript. All authors read and approved to the final manuscript.

## Pre-publication history

The pre-publication history for this paper can be accessed here:

http://www.biomedcentral.com/1471-2407/9/341/prepub

## Supplementary Material

Additional file 1 **Table S1: Enrollment and Outcomes. Diagnostic accuracy of frozen section in patients undergoing CNB for suspect breast lesions**. The data provided represent a flow chart of the patients enrolled. Click here for file
