# Supplementary material for: The accuracy of frozen section analysis in ultrasound- guided core needle biopsy of breast lesions
Source: BMC Cancer. 2009 Sep 24;9:341. doi: 10.1186/1471-2407-9-341 (PMC2759967; doi:10.1186/1471-2407-9-341)
Supplement: Additional file 1 — Table S1: Enrollment and Outcomes. Diagnostic accuracy of frozen section in patients undergoing CNB for suspect breast lesions. The data provided represent a flow chart of the patients enrolled. [file 1471-2407-9-341-S1.doc]

**Table 3.** Enrollment and Outcomes. Diagnostic accuracy of frozen section in patients undergoing CNB for suspect breast lesions.

120 breast samples-

109 patients with simple CNB

11 patients with multiple CNB

61 CNB evaluated by paraffin section analysis

59 CNB evaluated by frozen section and paraffin section analysis

2 cases with non specific diagnosis by frozen section analysis

40 malignant finding by frozen section analysis

15 cases with correct benign diagnosis by frozen section analysis

40 cases with correct malignant diagnosis by frozen section analysis

2 cases with non specific diagnosis by frozen section - in definitve diagnosis benign

2 cases with incorrect diagnosis- false benign by frozen section analysis

17 benign finding by frozen section analysis

CNB= core needle biopsy
